# Choline and *Choline alphoscerate* Do Not Modulate Inflammatory Processes in the Rat Brain

**DOI:** 10.3390/nu9101084

**Published:** 2017-09-29

**Authors:** Seyed Khosrow Tayebati, Ilenia Martinelli, Michele Moruzzi, Francesco Amenta, Daniele Tomassoni

**Affiliations:** 1 School of Medicinal Sciences and Health Products, University of Camerino, 62032 Camerino, Italy; ilenia.martinelli@unicam.it (I.M.); michele.moruzzi@unicam.it (M.M.); francesco.amenta@unicam.it (F.A.); 2 School of Biosciences and Veterinary Medicine, University of Camerino, 62032 Camerino, Italy; daniele.tomassoni@unicam.it

**Keywords:** choline, *Choline alphoscerate*, acetylcholine, inflammatory markers, rat brain

## Abstract

Choline is involved in relevant neurochemical processes. In particular, it is the precursor and metabolite of acetylcholine (ACh). Choline is an essential component of different membrane phospholipids that are involved in intraneuronal signal transduction. On the other hand, cholinergic precursors are involved in ACh release and carry out a neuroprotective effect based on an anti-inflammatory action. Based on these findings, the present study was designed to evaluate the effects of choline and choline precursor (*Choline alphoscerate*, GPC) in the modulation of inflammatory processes in the rat brain. Male Wistar rats were intraperitoneally treated with 87 mg of choline chloride/kg/day (65 mg/kg/day of choline), and at choline-equivalent doses of GPC (150 mg/kg/day) and vehicle for two weeks. The brains were dissected and used for immunochemical and immunohistochemical analysis. Inflammatory cytokines (Interleukin-1β, IL-1β; Interleukin-6, IL-6 and Tumor Necrosis Factor-α, TNF-α) and endothelial adhesion molecules (Intercellular Adhesion Molecule, ICAM-1 and Vascular cell Adhesion Molecule, VCAM-1) were studied in the frontal cortex, hippocampus, and cerebellum. The results clearly demonstrated that treatment with choline or GPC did not affect the expression of the inflammatory markers in the different cerebral areas evaluated. Therefore, choline and GPC did not stimulate the inflammatory processes that we assessed in this study.

## 1. Introduction

Choline is an essential nutrient in the health and development of humans [1,2]. It is a precursor of the neurotransmitter acetylcholine (ACh) and an agonist on ACh receptors [3]. It is involved in the transport of cholesterol and fats across cell membranes (lipoproteins) and induces methyl-group metabolism (plasmatic homocysteine reduction) [4]. Choline treatment stimulates ACh synthesis and release, increasing cholinergic transmission [5].

ACh and choline are fundamental for memory and cognitive functions [6,7,8,9]. With aging, a loss of short-term memory is related to a decrease of brain cholinergic neurons, of ACh synthesis and release as well as a compromised function of its receptors [10]. Some of these aspects are involved in the pathophysiology of Alzheimer’s disease (AD), where the brain cholinergic neurons become more vulnerable and prone to degeneration, because of defective cell membrane mechanisms. Consequently, a decreased availability of choline and increased breakdown of phosphatidylcholine have been reported as relevant conditions for AD pathophysiology [11,12].

Choline and the cholinergic precursors are important in the preservation of the structural integrity of cell membranes [13,14]. Cytidine-5′-diphosphocholine (CDP) and l-alpha-glycerylphosphorylcholine (*Choline alphoscerate*), (GPC) represent the choline precursors proposed as an alternative to choline, with larger clinical evidence in the treatment of sequelae of cerebrovascular accidents and cognitive decline in aging and AD [15,16,17,18]. 

CDP and GPC are both natural, water-soluble and show similar effects in different conditions [19]. CDP and GPC, orally administrated, cross the blood-brain barrier and are incorporated into the phospholipid fraction of the neuronal plasma membrane and in microsomes [20,21,22].

CDP supplementation induces the synthesis of structural phospholipids in neuronal membranes, increases cerebral metabolism, and enhances various neurotransmitter systems [14] such as the cholinergic and dopaminergic one [22,23]. CDP acts as a stimulant and neuroprotective factor for cultured dopaminergic neurons [24]. CDP and GPC increase the release and bioavailability of ACh in the hippocampus of rats, and improves attention and memory in patients with vascular dementia (VaD) [25], indicating that they may represent cholinergic neurotransmission enhancing compounds [17,18]. For these reasons, CDP supplementation protects against the development of memory deficits in aging rats [26], and its use has shifted from the treatment of acute, to chronic cerebrovascular disorders. CDP has also been occasionally prescribed as an adjuvant to l-Dopa treatment in Parkinson’s disease patients [17]. In some studies, CDP was used in the treatment of primary degenerative dementia or of mild forms of primary cognitive deterioration in elderly patients [27].

GPC is probably, among the choline precursors, the most effective in enhancing in vivo ACh release [15,28]. According to this evidence, the cognitive domain of AD and VaD patients has also been investigated [25,29,30]. Preclinical studies have demonstrated that GPC facilitates learning and memory, improves brain-transduction mechanisms, and decreases age-dependent structural changes occurring in the rat frontal cortex and hippocampus [15,31,32,33]. Moreover, the compound contributes to anabolic processes responsible for membrane phospholipids and glycerolipids synthesis, positively influencing membrane fluidity [34]. GPC has also been demonstrated to improve cognitive deficit in experimental models of the aging brain [35,36] and to reverse mnemonic deficits induced by scopolamine administration [15,37].

Choline, and GPC were proposed as potential neuroprotective agents for different pathological and/or not pathological conditions based on inflammatory processes [14,38]. Inflammatory endothelial cell activation and leukocyte endothelial interactions can be positively influenced by cholinergic mediators [39,40]. A beneficial cholinergic anti-inflammatory effect on endothelial function was shown through the activation of anti-inflammatory neuro-immunological mechanisms which modulate the innate immune response by limiting the pro-inflammatory process, thereby minimizing tissue injury [41,42]. 

Based on the above evidence, the present study was designed to further investigate the effects of choline and GPC in the modulation of inflammatory processes in the rat brain by analyzing their activity on cytokines [interleukin-1β (IL-1β), interleukin-6 (IL-6), and tumor necrosis factor alpha (TNF-α)] and vascular adhesion molecules.

## 2. Materials and Methods

### 2.1. Animals, Tissue Processing and Treatment

Male Wistar rats (220 ± 20 g; *n* = 24) were treated i.p. with 87 mg/kg/day of choline chloride (65 mg/kg/day of choline, *n* = 8), and at choline-equivalent doses of GPC (150 mg/kg/day, *n* = 8) and vehicle (water used for injectable solutions, *n* = 8) for 2 weeks. Animals were handled as per the internationally accepted principles for care of laboratory animals (European Community Council Directive 86/609, O.J. No. L358, 18 December 1986). After treatment, the animals were anesthetized with pentobarbital sodium (50 mg/kg, i.p.) and sacrificed by decapitation, the skull was opened and the brain was removed. The left hemisphere was processed for immunohistochemistry analysis using a fixative solution, containing 4% paraformaldehyde in 0.1 M phosphate buffer (pH 7.4) at 25 °C. After fixation at room temperature, the samples were gradually dehydrated in ethanol and embedded in paraffin. 

From the right hemisphere frontal cortex, the hippocampus and cerebellum were dissected and processed for Western blot analysis.

### 2.2. Western Blot Analysis 

Samples (0.1 ± 0.02 g) were homogenized in a Mixer Mill MM300 (Qiagen, Hilden, Germany) with 800 μL of 0.1 M phosphate buffer saline (PBS) pH 7.4, 0.1% IGEPAL CA-630, 1 mM CaCl_2_, 1 mM MgCl_2_, 0.1% NaN_3_, 1 mM phenyl-methyl-sulphonil-fluoride (PMSF), aprotinin, and 1 mM sodium ortovanadate. Next, after two centrifugations at 13,000 rpm (10 min at 4 °C), aliquots of the supernatant were used for protein assay against a standard of bovine serum albumin (BSA) using a BIO-RAD protein assay (Cat. No. 500-0001, BIO-RAD, Munich, Germany). Equal amount of proteins (40 μg) were separated by sodium dodecyl sulfate polyacrylamide gel electrophoresis and transferred to nitrocellulose membrane by electroblotting in the Towbin buffer [43]. Transblotted membranes were incubated with polyclonal antibodies as detailed in Table 1. The specificity of immune reaction was assessed using antibodies pre-adsorbed with peptides used for generating them. Blots were then washed in PBS + TritonX-100 (PBS-T) and incubated with the horseradish-peroxidase-linked secondary antibody (donkey anti-goat IgG Cat. No. A50-101P, goat anti-rabbit IgG Cat. A120-101P or goat anti-mouse IgG Cat. No. A90-116P, BETHYL Laboratories, Inc., Montgomery, TX, USA) at a dilution of 1:5000 for 60 min at room temperature. Positive bands were visualized by an enhanced chemiluminescence system (Lite Ablot^®^ plus, Cat. EMP 011005, Euroclone, Life Sciences Division, Siziano, Italy). To normalize protein loadings, membranes were stripped and incubated with a mouse monoclonal anti-β-actin antibody (clone AC-74, Cat. No. A2228, Sigma-Aldrich Co., St. Louis, MO, USA) at a dilution of 1:3000 in PBS-T overnight at 4 °C. Band intensities were measured by densitometry with Nikon Imaging Software (NIS Elements) (Nikon, Florence, Italy). 

### 2.3. Immunohistochemistry 

Sagittal sections of the brain 10 μm thick were cut using a microtome and collected on Superfrost plus slides. The brain sections were exposed to different antibodies of inflammatory cytokines (IL1-β, IL-6, and TNF-α) and endothelial inflammatory markers (Intercellular Adhesion Molecule, ICAM-1 and Vascular cell Adhesion Molecule, VCAM-1). 

Antibodies were diluted in PBS-T 0.3% (200 μL per section). Optimal antibody concentration was established in a series of preliminary experiments. Slides were incubated overnight at 4 °C with primary antibodies (Table 1). Non-specific binding of IgGs was prevented by incubating them with BSA 3% in PBS-T for 1 h. The product of immune reaction was then revealed by incubating slides for 30 min at 25 °C with the specific biotinylated secondary IgGs (donkey anti-goat IgG Cat. No. A50-101B, goat anti-rabbit IgG, Cat. A120-101B or goat anti-mouse IgG, Cat. No. A90-116B, BETHYL Laboratories, Inc.) of anti-goat, anti-mouse, and anti-rabbit diluted 1:200 in PBS-T. The immune reaction was then revealed with diaminobenzidine (0.05% 3-3′-diaminobenzidine dissolved in 0.1% H_2_O_2_) as a substrate. Slides were then washed, mounted on cover slips and viewed under a light microscope. Some sections were incubated with a non-immune serum instead of a primary antibody to assess the background of immunostaining. Before dehydration in ethanol, sections were also counterstained with haematoxylin.

### 2.4. Data Analysis

The averages of different parameters investigated were calculated from single animal data, and group means ± SEM were then derived from mean single animal values. The significance of the differences between the averages was analyzed by analysis of variance (ANOVA) followed by the Newman-Keuls multiple range test. Significance level was set for *p* < 0.05 to evaluate difference between studied groups.

## 3. Results

At the end of treatment, the body weight values were similar in different groups (vehicle 253.66 ± 4.12 g; choline treated 244.5 ± 3.4 g *p* = 0.21 vs. vehicle; GPC treated 261.5 ± 5.1 g *p* = 0.44 vs. vehicle). Brain weight values were not significantly different in the three animal groups (vehicle 1.81 ± 0.02 g; choline treated 1.85 ± 0.04 g *p* = 0.41 vs. vehicle; GPC treated 1.81 ± 0.03 g *p* = 0.19 vs. vehicle).

### 3.1. Immunochemical Analysis

**Figure 1:**
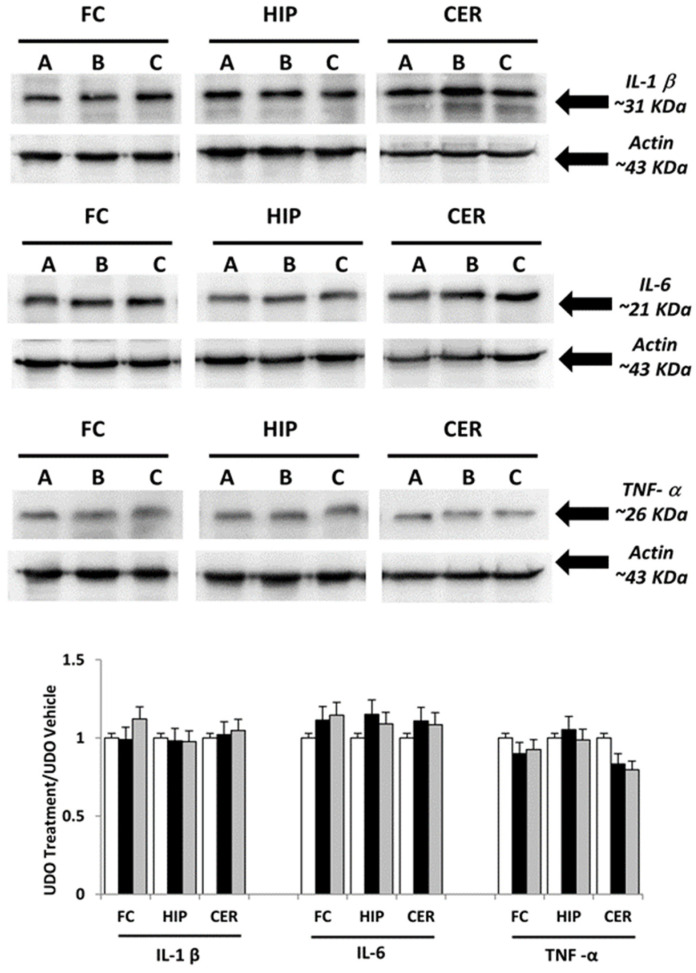
Immunochemical analysis of the frontal cortex (FC), hippocampus (HIP) and cerebellum (CER) processed with different antibodies (anti-IL-1β, anti-IL-6 and anti-TNF-α). A: vehicle; B: choline-treated; and C: L-alpha-glycerylphosphorylcholine (GPC)-treated. The densitometric analysis of bands are expressed as ratio between optical density of protein and reference protein (β-actin) where the value of vehicle is set as 1. Data are the mean ± SD of three different experiments. White bar: A vehicle; Black bar: B Choline-treated; Gray bar: C GPC-treated.

**Figure 2:**
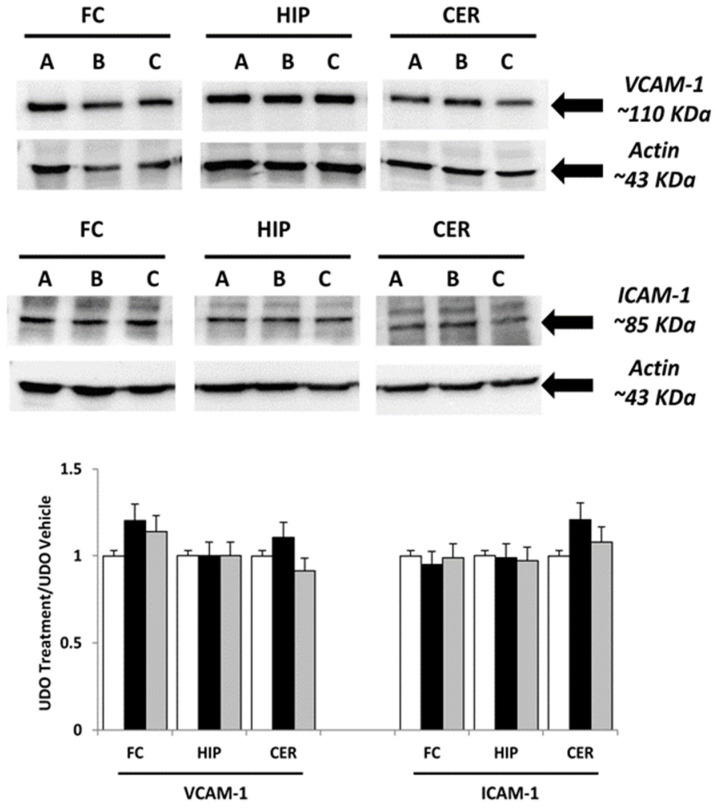
Immunochemical analysis of the frontal cortex (FC), hippocampus (HIP) and cerebellum (CER) processed with different antibodies (anti-VCAM-1 and anti-ICAM-1). A: vehicle; B: choline-treated; and C: GPC-treated. The densitometric analysis of bands are expressed as ratio between the optical density of protein and reference protein (β-actin) where the value of vehicle is set at 1. Data are the mean ± SD of three different experiments. White bar: A vehicle; Black bar: B Choline-treated; Gray bar: C GPC-treated.

Immunochemical analysis was performed on samples of brain areas of animals treated with choline, and at choline-equivalent doses of GPC or vehicle. The interleukins IL-1β, IL-6, TNF-α and adhesion molecules ICAM-1 and VCAM-1 were evaluated. In different areas, the analysis revealed a similar pattern of bands at 31 kDa for IL-1β, 21 kDa for IL-6 and 26 kDa for TNF-α (Figure 1), 85 kDa to ICAM-1, 110 kDa for VCAM-1, approximately (Figure 2). Evaluation of the different bands was made for different brain areas (frontal cortex, hippocampus and cerebellum) referring to the density of β-actin reference proteins. 

Western blot analysis of IL-1β and IL-6 bands demonstrated that the treatment with GPC or choline did not change the expression of these pro-inflammatory factors (Figure 1). A slight, but not significant effect on TNF- α was observed in the brain areas of animals treated with choline and GPC (Figure 1). 

The expression of ICAM-1 was lower when compared to the VCAM-1 in the different brain areas (Figure 2). Adhesion molecule VCAM-1 expression was not significantly decreased in the rat hippocampus after treatment with choline (Figure 2). Treatment with GPC did not change VCAM-1 expression. Similarly, treatment with GPC or the treatment with choline did not affect ICAM-1 expression in all of the examined tissues (Figure 2). 

### 3.2. Immunohistochemical Analysis

**Figure 3:**
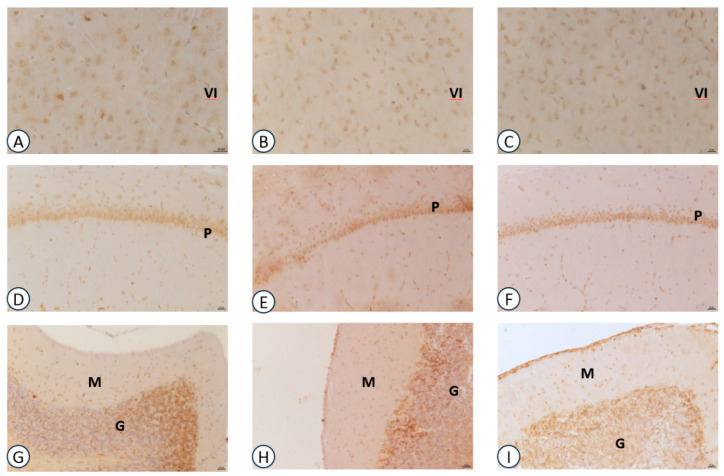
Sections of the frontal cortex (**A**–**C**), hippocampus (**D**–**F**), cerebellum (**G**–**I**) processed for IL-1β immunohistochemistry (**A**,**D**,**G**): vehicle: (**B**,**E**,**H**): choline-treated; (**C**,**F**,**I**): GPC-treated. VI: sixth layer of frontal cortex; P: pyramidal neurons of CA1 subfield of hippocampus; M: molecular layer of cerebellum; G: granular layer of cerebellum. Calibration bar: 25 μm.

Sections processed for IL-1β immunohistochemistry revealed dark-brown immunoreaction throughout the brain areas investigated. The immunoreaction was localized in the extracellular spaces around the body of neurons in all animal groups investigated. No reaction was detected within the perikaryon of pyramidal neurons of the frontal cortex (Figure 3A–C) and hippocampus (Figure 3D–F). IL-1β positive neurons were detected in the granular layer of cerebellar cortex (Figure 3G–I). In the frontal cortex, no difference in IL-1β expression was observed between the choline-, GPC-treated and control animals (Figure 3). 

**Figure 4:**
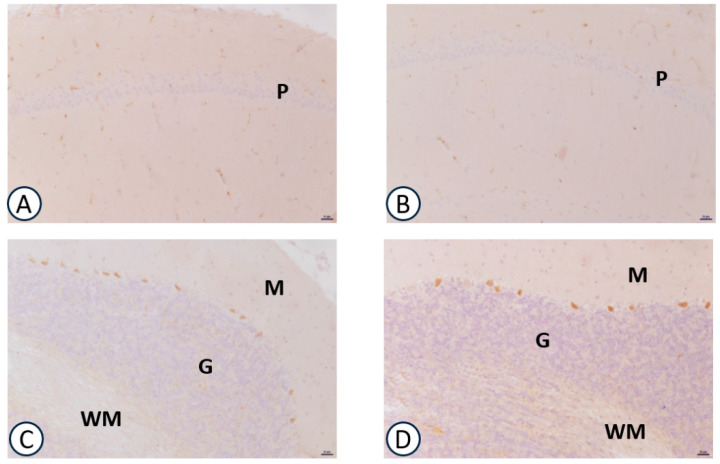
Sections of the CA1 subfields of hippocampus (**A**,**B**), cerebellum (**C**,**D**) processed for TNF-α immunohistochemistry. A, C: vehicle; B, D: GPC-treated. P: pyramidal neurons of CA1 subfield of hippocampus; M: molecular layer of cerebellum; G: granular layer of cerebellum; WM: white matter of cerebellum. Calibration bar: 25 μm.

A weak immunoreaction for IL-6 was observed in the different brain areas investigated without change for different experimental groups (data not shown). The immunohistochemistry for TNF-α was mainly localized in the hippocampus. The immunoreaction was slightly decreased in the CA1 subfield of the hippocampus of GPC-treated animals (Figure 4B), but not in the frontal cortex (data not shown). Treatment with choline did not change the TNF-α expression in different examined cerebral areas (data not shown).

**Figure 5:**
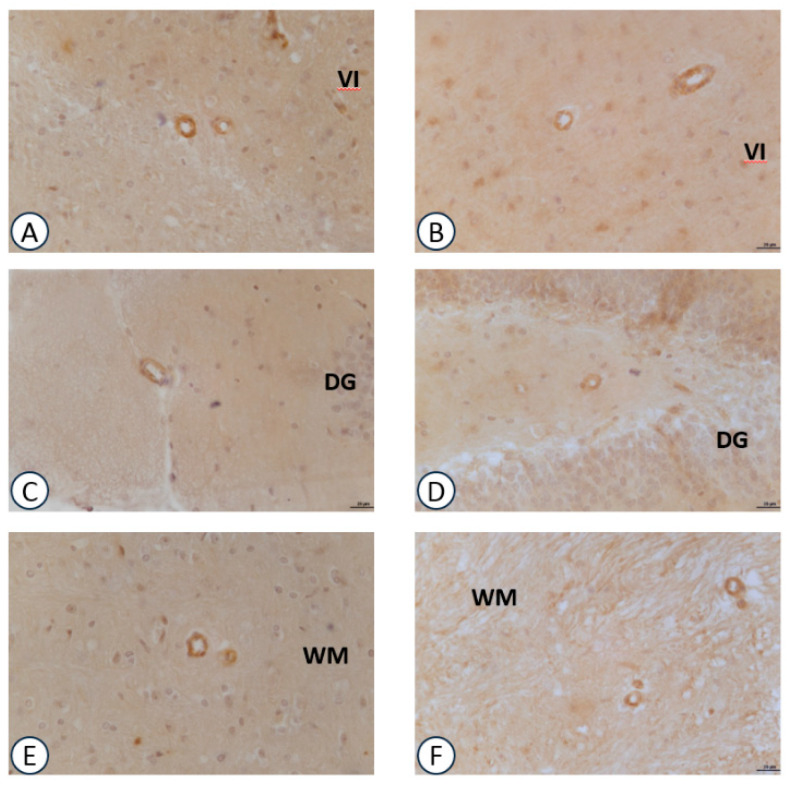
Sections of the frontal cortex (**A**,**B**), dentate gyrus (**C**,**D**), cerebellum (**E**,**F**) processed for VCAM-1 immunohistochemistry. (**A**,**C**,**E**): vehicle; (**B**,**D**,**F**): GPC-treated. VI: sixth layer of frontal cortex; DG: dentate gyrus; M: molecular layer of cerebellum; WM: white matter of cerebellum. Calibration bar: 25 μm.

**Figure 6:**
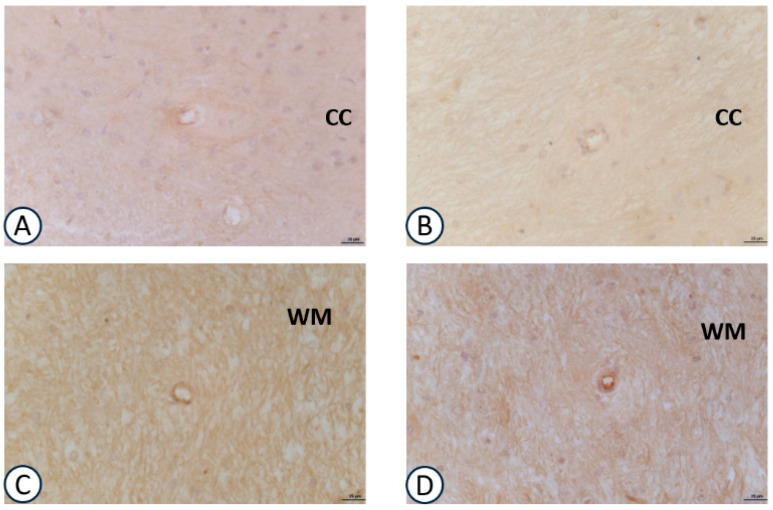
Sections of the frontal cortex (**A**,**B**), cerebellum (**C**,**D**) processed for ICAM-1 immunohistochemistry. (**A**,**C**): vehicle; (**B**,**D**): GPC-treated. CC: corpus callosum of frontal cortex; WM: white matter of cerebellum. Calibration bar: 25 μm.

Immunoreactivity for VCAM-1 in the intracerebral arteries (Figure 5) was more expressed when compared to the other adhesion molecule ICAM-1 (Figure 6). The immunoreaction was localized at the endothelial level and at the level of the muscular layer of the small sized (diameter range <50 μm) intracerebral arteries. Both treatment with GPC (Figure 5B,D,F) and choline (data not shown) did not modify the immunoreactions for VCAM-1. The same pattern was observed for ICAM-1 (Figure 6B,D).

## 4. Discussion and Conclusions

Choline and choline precursors (phosphatidylcholine, GPC, CDP-choline, sphingosylphosphorylcholine and lysophosphatidylcholine) represent molecules that can potentially increase ACh release and improve the integrity of cell membranes [13,14,15,16,44,45,46,47]. However, the decrease of ACh and the breakdown of cell membranes resulting from several pathological processes may evolve in nerve cell injury and neurological disorders [18,48].

On the other hand, ACh interacts with innate immune cells that express the nicotinic ACh receptor subunit α7 (α7nAChR). The activation of intracellular α7nAChR signal transduction suppressed the transcription of pro-inflammatory genes [41,42] and endothelial cell activation [49]. 

Local administration of some choline procursors (e.g., CDP-choline, CDP) reduced tissue edema and TNF-production in a carrageenan-induced inflammatory pain model mediated via α7nAChRs [50]. Several studies have described the protective effect of CDP on microvascular permeability during experimental endotoxemia; however, this does not affect leukocyte adherence [51]. Tissue pro-inflammatory cytokines (IL-1β, IL-6 and TNF-α) were also reduced by CDP treatment [52]. Moreover, choline deficiency enhanced endotoxin-induced hepatotoxicity [53]. In fact, intravenous choline treatment mitigated endotoxin-induced organ injury and the increment of circulating TNF-α in dogs [54], and improved survival in mice with endotoxin and septic shock [55]. 

High concentrations of choline (400 µM in dogs and rats) can activate nAChR [3] on circulating immune cells (i.e., monocytes, lymphocytes, macrophages) and inhibit the release of pro-inflammatory cytokines in response to endotoxin [55]. The fact that choline suppresses endotoxin-induced cytokine release from monocyte/lymphocytes and/or macrophages is directly supported by experimental data where choline, at 1–50 mM concentrations inhibited the release of TNF-α from macrophages [55,56]. Other choline procursors and GPC modulated astroglial proliferation in both in vitro and in vivo studies suggested a possible protective effect on the brain [44,45,46,47].

On the basis of these data, the present study evaluated the effects of choline and GPC treatments on inflammatory markers in normal brain conditions. The obtained results highlighted that in the basal conditions, choline and GPC did not modulate the expression of the pro-inflammatory cytokines and endothelial adhesion molecules that were tested. Hence, these treatments did not involve inflammatory activation pathways by these molecules at the level of neurons and intracerebral arteries. In addition, it appears that they do not have any anti-inflammatory effects on these conditions. 

Therefore, the data suggested that although the use of choline and *Choline alphoscerate* increased ACh release and modulated the cholinergic system/dopaminergic system [13,14,15,22], it did not modify the cerebral inflammatory status. The modulator effect of ACh on inflammatory processes is documented, and it is known that like the peripheral response, ACh exerts a neuroprotective effect through the cholinergic anti-inflammatory pathway in the brain [57]. Other studies have demonstrated that nicotine can suppress a lipopolisaccarides (LPS)-induced release of TNF-α in murine microglial cells via α7nAChR, and that this effect can be inhibited by a selective α7 antagonist [58].

In vesicular acetylcholine transporter (VAChT) knock down-mice, long-term VAChT deficiency exacerbates acute systemic and cerebral inflammation, as well as promotes neural activation and the concomitant sickness behavior induced by LPS administration [59]. The authors proposed that bidirectional communication (mainly between glutamatergic neurons and glial cells) led to an ACh release by astrocytes [60]; this ACh in turn binds to α7nAChR located in the microglia, thus allowing the activation of the cholinergic anti-inflammatory pathway [58]. This mechanism may be defective in VAChT knock down-mice, and this problem may perpetuate the inflammatory profile and intensify sickness behavior after LPS exposure. 

Previous studies on the effects of GPC on neuroinflammation have demonstrated that in pathological conditions (e.g., hypertension, edema), the compound had an anti-inflammatory effect, most likely due to the increase in ACh levels. In fact, in the animal model of hypertension, GPC treatment decreased astrogliosis reaction and the expression of adhesion molecules [44,45,46,47]. Conversely, in normal conditions, although the GPC [13,14] and choline [61] increased ACh release, it did not modulate the release of cytokines and expression of vascular adhesion molecules. Without specific pro-inflammatory events, the administration of choline and GPC and the consequent increase of ACh [13,14,61], did not modulate the inflammatory pathways through microglia cells activation. 

In conclusion, choline precursors contribute to stimulate cholinergic and monoaminergic neurotransmission [13,14] and, in our experimental conditions, do not activate specific molecules involved in the modulation of inflammatory processes. However, other studies may be necessary to investigate the possible anti-inflammatory properties of choline precursors in pre-clinical and clinical settings. 

## Figures and Tables

**Table 1:** The primary antibodies using for Western blot (WB) and immunohistochemistry (IHC).

Primary Antibody	Clone	Host Animal	Company	Cat. No.	Dilution for WB	Dilution for IHC
IL-1β	(H-153)	Rabbit	Santa Cruz Biotechnology, Inc. (Santa Cruz, CA, USA)	sc-7884	1:200	1:50
IL-6	(M-19)	Goat	Santa Cruz Biotechnology, Inc.	sc-1265	1:200	1:50
TNF-α	(52B83)	Mouse	Santa Cruz Biotechnology, Inc.	sc-52746	1:500	1:250
VCAM-1	(H-276)	Rabbit	Santa Cruz Biotechnology, Inc.	sc-8304	1:500	1:50
ICAM-1	(G-5)	Mouse	Santa Cruz Biotechnology, Inc.	sc-8439	1:500	1:50
β-actin	(AC-74)	Mouse	Sigma-Aldrich Co. (Saint Louis, MO, USA)	A2228	1:3000	-
