# Correction: Tayebati et al. Choline and *Choline alphoscerate* Do Not Modulate Inflammatory Processes in the Rat Brain. *Nutrients* 2017, *9*, 1084

**DOI:** 10.3390/nu16152462

**Published:** 2024-07-29

**Authors:** Seyed Khosrow Tayebati, Ilenia Martinelli, Michele Moruzzi, Francesco Amenta, Daniele Tomassoni

**Affiliations:** 1 School of Medicinal Sciences and Health Products, University of Camerino, 62032 Camerino, Italy; ilenia.martinelli@unicam.it (I.M.); michele.moruzzi@unicam.it (M.M.); francesco.amenta@unicam.it (F.A.); 2 School of Biosciences and Veterinary Medicine, University of Camerino, 62032 Camerino, Italy; daniele.tomassoni@unicam.it

For the original publication [1] and upon request from the scientific community, the authors have not been able to provide the original data used to assemble Figure 1 and Figure 2. Thus, the authors have repeated the experiment and confirmed the original results. Figure 1 and Figure 2 have been accordingly updated. 

A correction has been made to the second sentence in the last paragraph of Section 3.1, which now reads as follows:

Adhesion molecule VCAM-1 expression was not significantly decreased in the rat hippocampus after treatment with choline (Figure 2).

The authors apologize for any inconvenience caused and state that the scientific conclusions are unaffected. This correction was approved by the Academic Editor. The original publication has also been updated.

## Figures and Tables

**Figure 1:**
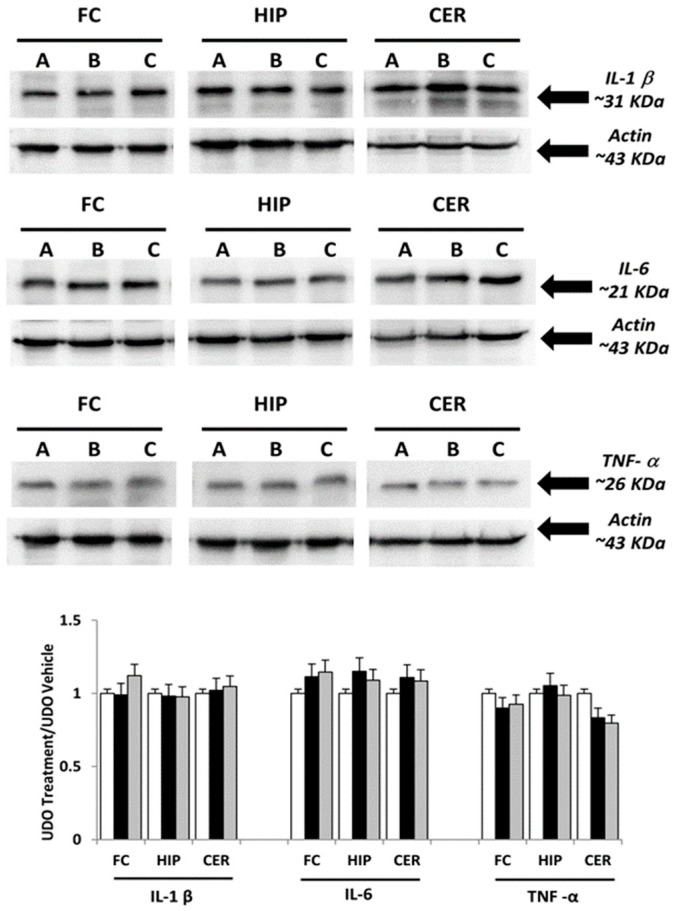
Immunochemical analysis of the frontal cortex (FC), hippocampus (HIP) and cerebellum (CER) processed with different antibodies (anti-IL-1β, anti-IL-6 and anti-TNF-α). A: vehicle; B: choline-treated; and C: L-alpha-glycerylphosphorylcholine (GPC)-treated. The densitometric analysis of bands are expressed as ratio between optical density of protein and reference protein (β-actin) where the value of vehicle is set as 1. Data are the mean ± SD of three different experiments. White bar: A vehicle; Black bar: B Choline-treated; Gray bar: C GPC-treated.

**Figure 2:**
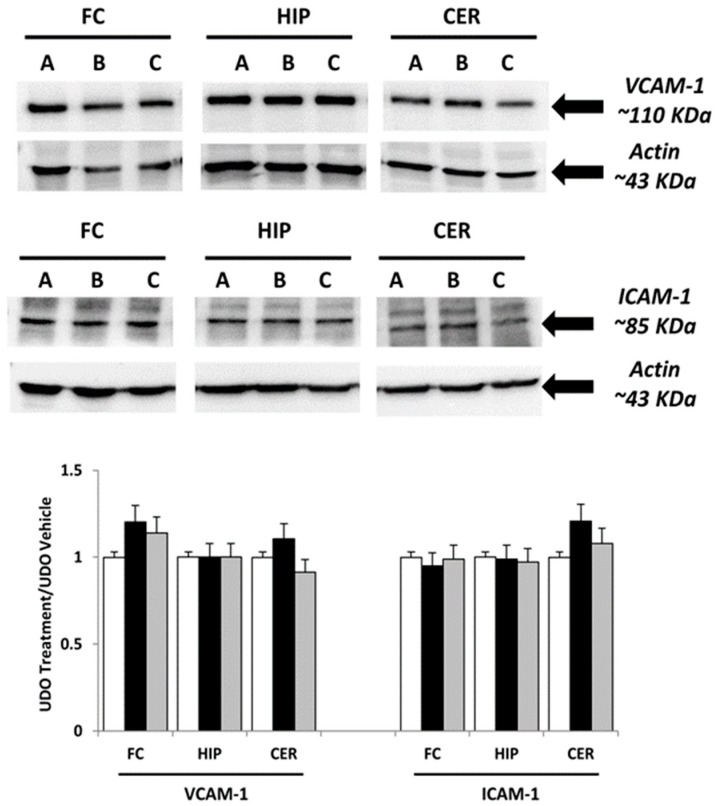
Immunochemical analysis of the frontal cortex (FC), hippocampus (HIP) and cerebellum (CER) processed with different antibodies (anti-VCAM-1 and anti-ICAM-1). A: vehicle; B: choline-treated; and C: GPC-treated. The densitometric analysis of bands are expressed as ratio between the optical density of protein and reference protein (β-actin) where the value of vehicle is set at 1. Data are the mean ± SD of three different experiments. White bar: A vehicle; Black bar: B Choline-treated; Gray bar: C GPC-treated.
